# Fetale Alkoholspektrumstörungen – Diagnose, Prognose und Prävention

**DOI:** 10.1007/s00103-021-03329-6

**Published:** 2021-05-03

**Authors:** Judith E. Moder, Lisa K. Ordenewitz, Julia A. Schlüter, Tobias Weinmann, Philine Altebäumer, Jessica Jung, Florian Heinen, Mirjam N. Landgraf

**Affiliations:** 1 Deutsches FASD KOMPETENZZENTRUM Bayern, LMU Zentrum für Entwicklung und komplex chronisch kranke Kinder – iSPZ Hauner, Dr. von Haunersches Kinderspital, LMU Klinikum München, Lindwurmstraße 4, 80337 München, Deutschland; 2 Abteilung Neuropädiatrie, Entwicklungsneurologie und Sozialpädiatrie, LMU Zentrum für Entwicklung und komplex chronisch kranke Kinder – iSPZ Hauner, Dr. von Haunersches Kinderspital, LMU Klinikum München, München, Deutschland; 3 Institut und Poliklinik für Arbeits‑, Sozial- und Umweltmedizin, LMU Klinikum München, München, Deutschland

**Keywords:** FASD, Fetales Alkoholsyndrom, FAS, pFAS, ARND, FASD, Fetal alcohol syndrome, FAS, pFAS, ARND

## Abstract

Die Fetale Alkoholspektrumstörung ist eine der häufigsten bei Geburt bestehenden chronischen Erkrankungen, die zum Großteil nicht oder fehldiagnostiziert wird. Dies führt zu inadäquater, ineffektiver Förderung und Therapie der erkrankten Kinder sowie mangelnder Unterstützung der betroffenen Familien. Daraus resultiert nicht nur ein hohes Maß an Sekundärerkrankungen, sondern auch eine Einbuße in der Möglichkeit der Sekundär- und Tertiärprävention bei erkrankten Kindern und betroffenen Familien. Sekundär und Tertiärprävention sind jedoch bei richtiger und rechtzeitiger Diagnose möglich. Die Primärprävention im Bereich Alkoholkonsum in der Schwangerschaft und Fetale Alkoholspektrumstörung muss auch in Zukunft sowohl von medizinischer als auch politischer Seite strukturiert, interdisziplinär und wissenschaftlich basiert geplant und durchgeführt werden. Neben der Aufklärung der Allgemeinbevölkerung ist hierbei die Wissensvermittlung an ÄrztInnen und andere medizinisch-psychologisch-pädagogische Fachkräfte besonders relevant.

## Abstract (en)

Fetal alcohol spectrum disorder is one of the most common chronic diseases at birth. It is very often not or falsely diagnosed. This leads to inadequate, ineffective care and treatment of the affected children as well as to insufficient support of their families. The result is not only a high rate of secondary disorders, but it also leads to fewer opportunities for secondary and tertiary prevention for each child and family. With the right diagnosis these opportunities are guaranteed. The primary prevention in the field of alcohol consumption during pregnancy and fetal alcohol spectrum disorder should be planned and performed by the medical and political partners in a structured interdisciplinary and scientifically based way. Besides health promotion and education of the general population, the knowledge transfer to doctors and other medical, psychological, and pedagogical professionals is of particular relevance.

## Hintergrund

In Deutschland existiert eine alkoholpermissive Gesellschaft. Dadurch ist Alkohol eines unserer häufigsten „Umweltgifte“. Für den eigenen Alkoholkonsum und die Inkaufnahme möglicher Gesundheitsrisiken ist (auch wenn dies das Gesundheitssystem insgesamt belastet) jeder erwachsene Mensch selbst verantwortlich. Dies ändert sich jedoch bei mütterlichem Alkoholkonsum während der Schwangerschaft, da das ungeborene, „nicht mitredende“ Kind das Folgerisiko trägt. In der Präventionsarbeit sind die Aufklärung und Sensibilisierung werdender Eltern über die Gefahr der intrauterinen Alkoholschädigung ihres Kindes und die Unterstützung in Hinsicht auf eine eigenverantwortliche Entscheidung *gegen* den Alkoholkonsum besonders relevant. Ein oktroyiertes Verbot widerspricht (zu Recht) dem Eigenbild vieler moderner Frauen und führt daher nicht zum Erfolg, also nicht zum Alkoholverzicht und damit zum Schutz des Ungeborenen. Zu beachten ist auch, dass der mütterliche Alkoholkonsum nicht das Problem der einzelnen Frau, sondern ein gesellschaftliches Problem ist. Es existieren nicht nur zahlreiche psychische und soziale Gründe, weshalb Frauen im gebärfähigen Alter Alkohol trinken. Das soziale Umfeld fordert (insbesondere bei sozialen Festivitäten und Geselligkeiten) sogar oft eine Rechtfertigung für den Alkoholverzicht. Dies ist bei anderen Substanzen nicht der Fall und daraus folgt, dass hier ein gesellschaftliches Umdenken notwendig ist.

Im vorliegenden Artikel soll eine Übersicht über epidemiologische Daten zu intrauteriner Alkoholexposition und zur Fetalen Alkoholspektrumstörung (Fetal Alcohol Spectrum Disorder, FASD), die Diagnose und Prognose der FASD sowie über Präventionsansätze gegeben werden.

## Epidemiologie

Laut der Studie „Gesundheit in Deutschland Aktuell“ geben ca. 20 % aller Frauen einen moderaten und ca. 8 % einen riskanten Alkoholkonsum während der Schwangerschaft an [1]. Auch Rauschtrinken (≥ 5 Getränke pro Gelegenheit) kommt während der Schwangerschaft vor: bei 12 % der Frauen seltener als einmal pro Monat, bei knapp 4 % jeden Monat und bei 0,1 % mindestens jede Woche.

Alkohol gelangt ungehindert über die Plazenta in den embryofetalen Kreislauf und kann die Entwicklung aller Organe negativ beeinflussen. Besonders vulnerabel während der gesamten Schwangerschaft ist das sich entwickelnde kindliche Gehirn.

Führt die intrauterine Alkoholexposition zu einer toxischen Gehirnschädigung, zeigt das Kind nach der Geburt Auffälligkeiten in der Entwicklung, Kognition und im Verhalten sowie teilweise auch Wachstumsdefizite und faziale Anomalien. Diese Symptome werden im Störungsbild FASD zusammengefasst [2].

In Deutschland existiert keine flächendeckende Prävalenzstudie zur FASD. Kraus et al. schätzen anhand von statistischen Daten zu Alkoholkonsum in der Schwangerschaft und dem damit verbundenen Risiko der Entstehung einer FASD die Inzidenz auf 1,77 Kinder pro 100 Lebendgeburten [3]. Damit ist die FASD die häufigste, bei Geburt bestehende, chronische Erkrankung.

## Diagnose

Die Fetale Alkoholspektrumstörung (FASD) ist der Oberbegriff für folgende Störungsbilder: das Fetale Alkoholsyndrom (FAS), das partielle Fetale Alkoholsyndrom (pFAS) und die alkoholbedingte entwicklungsneurologische Störung (Alcohol-related Neurodevelopmental Disorder; ARND).

Für die Diagnose der verschiedenen FASD können die Kriterien und Empfehlungen der S3-Leitlinie herangezogen werden [4].

Im Folgenden werden diese Kriterien stichpunktartig aufgeführt.

### Die 4 diagnostischen Säulen des Fetalen Alkoholsyndroms (FAS)

**Figure 1:**
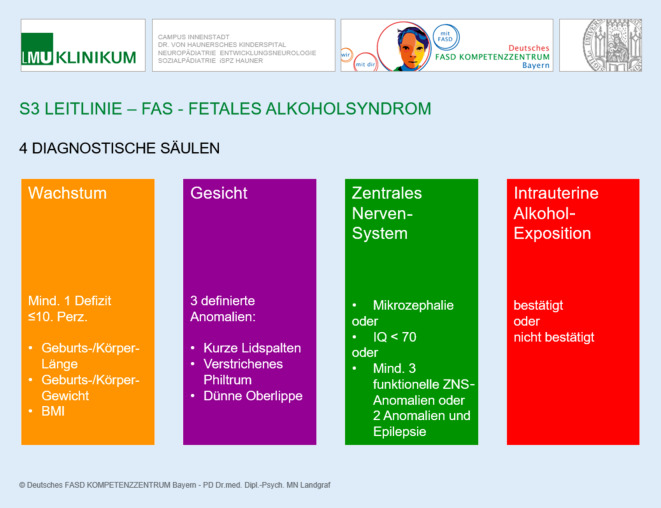

1. Wachstumsauffälligkeiten
2. Faziale Auffälligkeiten
3. ZNS-Auffälligkeiten
4. Intrauterine Alkoholexposition (Abb. 1)

Zu 1) Wachstumsauffälligkeiten sind definiert als:

- Geburts- oder Körpergewicht ≤ 10. Perzentile und/oder
- Geburts- oder Körperlänge ≤ 10. Perzentile und/oder
- Body-Mass-Index ≤ 10. Perzentile.

Die gemessenen Körpermaße sollten an das Gestationsalter, Alter des Kindes und Geschlecht adaptiert werden. Wachstumsdefizite können beim FAS zu jedem Zeitpunkt auftreten und als Diagnosekriterium gelten – ein ausgeprägtes Kreuzen mehrerer Perzentilen ist für FAS jedoch ungewöhnlich und sollte zu differenzialdiagnostischen Überlegungen führen (z. B. pränatale Mangelzustände, genetische Syndrome, chronische Erkrankungen, Malabsorption, Mangelernährung oder Vernachlässigung).

Zu 2) Zur Erfüllung des Kriteriums „faziale Auffälligkeiten“ sollen alle 3 fazialen Anomalien vorhanden sein:

- kurze Lidspalten (≤ 3. Perzentile bzw. mind. 2 Standardabweichungen unter der Norm),
- verstrichenes Philtrum (Rang 4 oder 5 auf dem Lip-Philtrum-Guide),
- schmale Oberlippe (Rang 4 oder 5 auf dem Lip-Philtrum-Guide).

Die Lidspaltenlänge kann mittels eines durchsichtigen, flexiblen Lineals direkt oder auf einer Fotografie mit Referenzmaßstab (z. B. 1 cm großer, auf die Stirn geklebter Punkt) gemessen und in die verfügbaren Perzentilenkurven eingetragen werden [4].

**Figure 2:**
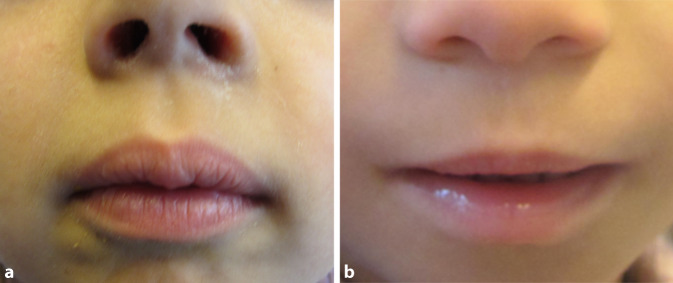

Die Oberlippe und das Philtrum können anhand des Lip-Philtrum-Guides von Astley et al. [5] quantitativ eingeordnet werden. Dabei gelten Messungen mit 4 und mit 5 von 5 Punkten auf der Skala als pathologisch. In der Abb. 2a sind die Oberlippe und das Philtrum eines gesunden, in Abb. 2b eines an FAS erkrankten Kindes dargestellt.

Zu 3) Zur Erfüllung der diagnostischen Säule „ZNS-Auffälligkeiten“ sollte eine Mikrozephalie (≤ 10. Perzentile) vorliegen oder das folgende Kriterium „funktionelle ZNS-Auffälligkeiten“ erfüllt sein.

Zur Erfüllung des Kriteriums „funktionelle ZNS-Auffälligkeiten“ sollte mindestens 1 der folgenden Auffälligkeiten zutreffen, die nicht adäquat für das Alter ist und nicht allein durch den familiären Hintergrund oder das soziale Umfeld erklärt werden kann:

- globale Intelligenzminderung mindestens 2 Standardabweichungen unterhalb der Norm oder signifikante kombinierte Entwicklungsverzögerung bei Kindern unter 2 Jahren,
- Leistung mindestens 2 Standardabweichungen unterhalb der Norm in mindestens 3 der folgenden Bereiche oder in mindestens 2 der folgenden Bereiche in Kombination mit Epilepsie:
  Sprache,
  Feinmotorik,
  räumlich-visuelle Wahrnehmung oder räumlich-konstruktive Fähigkeiten,
  Lern- oder Merkfähigkeit,
  exekutive Funktionen,
  Rechenfertigkeiten,
  Aufmerksamkeit,
  soziale Fertigkeiten oder Verhalten.

Zu 4) Wenn Auffälligkeiten in den 3 übrigen diagnostischen Säulen bestehen, soll die Diagnose eines Fetalen Alkoholsyndroms auch *ohne *Bestätigung eines mütterlichen Alkoholkonsums während der Schwangerschaft gestellt werden.

### Die 3 diagnostischen Säulen des partiellen Fetalen Alkoholsyndroms (pFAS)

1. Faziale Auffälligkeiten
2. ZNS-Auffälligkeiten
3. Intrauterine Alkoholexposition

Die Wachstumsäule entfällt für die Diagnose des pFAS.

Zu 1) Für die Diagnose des pFAS sollen 2 der 3 oben beschriebenen fazialen Auffälligkeiten, dokumentiert zu einem beliebigen Zeitpunkt, vorhanden sein.

Zu 2) Zur Erfüllung des Kriteriums „ZNS-Auffälligkeiten“ sollen mind. 3 der folgenden Auffälligkeiten zutreffen, die nicht adäquat für das Alter sind und nicht allein durch den familiären Hintergrund oder das soziale Umfeld erklärt werden können:

- globale Intelligenzminderung (mind. 2 Standardabweichungen unter der Norm) oder signifikante kombinierte Entwicklungsverzögerung bei Kindern ≤ 2 Jahren,
- Epilepsie,
- Mikrozephalie ≤ 10. Perzentile.

Leistung mind. 2 Standardabweichungen unter der Norm in den Bereichen:

- Sprache,
- Fein‑/Grafomotorik oder grobmotorische Koordination,
- räumlich-visuelle Wahrnehmung oder räumlich-konstruktive Fähigkeiten,
- Lern- oder Merkfähigkeit,
- exekutive Funktionen,
- Rechenfertigkeiten,
- Aufmerksamkeit,
- soziale Fertigkeiten oder Verhalten.

Im Gegensatz zum FAS reicht beim pFAS das alleinige Vorliegen einer Mikrozephalie oder einer Intelligenzminderung für die Diagnose nicht aus. Alle mit Aufzählungspunkten versehenen Bereiche gelten als gleichwertig für die Diagnosestellung.

Zu 3) Die intrauterine Alkoholexposition sollte für die Diagnose eines pFAS bestätigt oder wahrscheinlich sein, um Über- bzw. Fehldiagnosen zu vermeiden.

### Die 2 diagnostischen Säulen der alkoholbedingten entwicklungsneurologischen Störung (ARND)

1. ZNS-Auffälligkeiten
2. Intrauterine Alkoholexposition

Die Säulen der Wachstums- und fazialen Auffälligkeiten entfallen.

Zu 1) Mindestens 3 ZNS-Auffälligkeiten, die denen vom pFAS (s. oben) entsprechen, sollen für die Diagnose der ARND vorliegen.

Zu 2) Wenn ZNS-Auffälligkeiten vorhanden sind, soll die Diagnose einer ARND bei bestätigtem mütterlichen Alkoholkonsum während der Schwangerschaft gestellt werden.

### Differenzialdiagnosen

Auch wenn die FASD im Vergleich zu anderen bei Geburt bestehenden Erkrankungen sehr viel häufiger ist, sollte immer auch kritisch differenzialdiagnostisch vorgegangen werden, um Fehldiagnosen beim Kind und eine mit der Diagnose potenziell einhergehende soziale Stigmatisierung der biologischen Mutter zu vermeiden. Aktuell liegt in Deutschland allerdings laut statistischen Schätzungen eine deutliche Unterdiagnose der FASD vor. Dies führt zu einem fehlenden oder fehlerhaften Krankheitskonzept und enthält damit den erkrankten Kindern eine adäquate Förderung und Therapie vor.

Eine ausführliche Liste mit möglichen Differenzialdiagnosen der FASD (darunter z. B. Wachstums-, genetische und psychiatrische Störungen) befindet sich, für jede diagnostische Säule separat dargestellt, in der S3-Leitlinie [4].

## Prognose

Die phänotypischen Auffälligkeiten der an FASD Erkrankten können sich im Jugend- und Erwachsenenalter reduzieren [6], die Funktionsbeeinträchtigungen des Gehirns persistieren jedoch. Vor allem die bei den meisten Menschen mit FASD vorliegende Exekutivfunktionsstörung führt zu signifikanten Schwierigkeiten bis hin zur Unfähigkeit, den Alltag zu strukturieren und selbstständig zu wohnen und zu arbeiten [6, 7].

Die ZNS-Funktionsdefizite bei PatientInnen mit FASD führen häufig zu äußerst frustrierenden Lebenserfahrungen und -verläufen. Psychiatrische Sekundärerkrankungen oder Komorbiditäten sind gemäß amerikanischen Studien extrem häufig. 50 % der Erwachsenen mit FASD leiden unter einer Depression, 55 % unter eigener Alkohol- oder Drogenabhängigkeit und 35 % weisen suizidales Verhalten auf [8, 9]. In einer deutschen Studie zeigte sich eine bessere Prognose mit einer Depression bei 5 % und Suchterkrankung bei 12 % der untersuchten Erwachsenen mit FAS [10].

Über protektive Faktoren ist insgesamt wenig bekannt. Am relevantesten zeigten sich bisher ein stabiles, förderndes Umfeld, eine frühe Diagnosestellung und ein gewaltfreies Aufwachsen [7, 11].

## Prävention

Wichtiges Ziel der Nationalen Strategie zur Drogen- und Suchtpolitik sowie klinischer Präventionsstrategien ist die Punktnüchternheit während der gesamten Schwangerschaft. Durch sie ist FASD vollständig vermeidbar [12]. Punktnüchternheit beschreibt einen vollständigen Verzicht auf Alkohol in bestimmten Situationen, wie z. B. in der Schwangerschaft. Grundlage dieses Zieles ist das Fehlen eines wissenschaftlich begründeten unbedenklichen Grenzwertes der Alkoholmenge, die in der Schwangerschaft konsumiert werden kann, ohne das ungeborene Kind zu schädigen [13]. Die EU-Strategie zur Unterstützung der Mitgliedstaaten zur Verringerung alkoholbedingter Schäden aus dem Jahr 2006 definiert den Schutz von Jugendlichen, Kindern und des Kindes im Mutterleib als einen EU-weit relevanten Schwerpunktbereich. Ein weiteres Ziel sind die Information, Aufklärung und Bewusstseinsbildung von Auswirkungen eines schädlichen und riskanten Alkoholkonsums [14]. Die Weiterentwicklung von FASD-Präventionsmodellen in Deutschland wurde im Rahmen der 2 Förderlinien des Bundesministeriums für Gesundheit (BMG) „Neue Präventionsansätze zur Vermeidung und Reduzierung von Suchtmittelkonsum in Schwangerschaft und Stillzeit“ (2010–2011) und „Verbreitung bewährter Präventionsansätze zur Vermeidung und Reduzierung von Suchtmittelkonsum in Schwangerschaft und Stillzeit“ (2012–2014) gefördert. Insgesamt wurden in der ersten Förderphase 7, in der zweiten Phase 3 dieser 7 Projekte weiterhin unterstützt und dabei extern evaluiert [15].

Präventionsmaßnahmen zur Vermeidung von FASD können, je nach angesprochener Zielgruppe, in universelle, selektive und indizierte Präventionsstrategien kategorisiert werden [16].

### Universelle Prävention

Die universelle Prävention richtet sich an die gesamte Bevölkerung oder Bevölkerungsgruppen – unabhängig von individuellen Risikofaktoren – und dient in erster Linie der Aufklärung und Sensibilisierung. In Bezug auf FASD umfasst diese Präventionsstrategie Maßnahmen, die frühzeitig und breitflächig über Alkoholkonsum während der Schwangerschaft und FASD informieren und Menschen für diese und damit verbundene Themen sensibilisieren.

Zu diesen Maßnahmen zählen im Wesentlichen massenmediale Aufklärungskampagnen, Warnhinweise auf Alkohol enthaltenden Getränkeflaschen (bisher nicht auf deutschen Produkten verpflichtend) und allgemeine Informationsbereitstellung über beispielsweise in Arztpraxen oder Kliniken ausliegende Flyer [17]. Die Bundeszentrale für gesundheitliche Aufklärung stellt auf ihrer Website Informationsmaterialen wie Faltblätter, Fachpublikationen und Beratungsbände für verschiedene Berufsgruppen zur Verfügung [18].

Die Ergebnisse zweier repräsentativer Erhebungen in der deutschen Bevölkerung bei Personen ab 14 Jahren aus den Jahren 2014 und 2017 ergaben hohe Zustimmungsraten zur Aussage, dass Alkohol während der Schwangerschaft generell problematisch ist: von 85 % der Befragten im Jahr 2014 und von 89 % im Jahr 2017. Die Aussage, dass der Alkoholkonsum einer schwangeren Frau im schlimmsten Fall zu schweren, lebenslangen Behinderungen des Kindes führen kann, hielten 2014 nur 56 % der Befragten für richtig. Der Anteil an Befragten, der dem zustimmte, erhöhte sich im Jahr 2017 auf 70 % [19, 20].

Als wichtige universell ausgerichtete Präventionsmaßnahme wird in der Internationalen Charta zur Prävention der FASD die Vermittlung genereller Information mittels evidenzbasierter Materialen über FASD in Schulen für beide Geschlechter genannt [21]. Die Schulpflicht schafft ein einzigartiges Setting, Jugendlichen bereits vor Eintreten einer Schwangerschaft und somit primärpräventiv Informationen über die gravierenden Folgen von Alkoholkonsum für das ungeborene Kind und die Auswirkungen auf das Leben von Betroffenen zu vermitteln. Diese Form der frühen FASD-Prävention ist aktuell nicht in den Lehrplänen allgemeinbildender Schulen integriert. Das BMG förderte von 2015 bis 2018 das Projekt „Schwanger? Dein Kind trinkt mit! Alkohol? Kein Schluck – kein Risiko!“ für SchülerInnen ab der 8. Schulstufe, welches von der Ärztlichen Gesellschaft zur Gesundheitsförderung e. V. entwickelt und im Rahmen von 1230 Veranstaltungen durchgeführt wurde. Die Ergebnisse der begleitenden clusterrandomisierten Wartekontrollgruppenstudie zeigten Effekte der Intervention auf das Wissen mit dem Umgang und über die Folgen von Alkoholkonsum in der Schwangerschaft. Der Anteil an SchülerInnen, welche angaben, dass Frauen in der gesamten Schwangerschaft keinen Alkohol trinken sollten, stieg in der Interventionsgruppe von 34,4 % auf 66,0 %. Die korrekte Antwort innerhalb der Kontrollgruppe blieb mit den erhobenen Werten von 38,4 % auf 41,7 % weitestgehend stabil. Wigwam Zero, ein weiteres vom Berliner Senat gefördertes Modellprojekt zur FASD-Prävention, hat in Zusammenarbeit mit der Fachstelle für Suchtprävention im Land Berlin das Medienpaket „Blau im Bauch?“ in Form eines jugendlichenadaptierten Schulungsvideos und dazugehöriger Lehr- und Diskussionsmaterialen für PädagogInnen und MultiplikatorInnen an Schulen entwickelt [22].

Zur universellen Prävention gehört neben der Aufklärung der Allgemeinbevölkerung und der Frauen im gebärfähigen Alter auch die Aufklärung werdender Väter. Alkohol- und/oder Drogenkonsum des Partners oder anderer enger Bezugspersonen birgt ein deutlich höheres Risiko für Alkoholkonsum der schwangeren Frau [23].

Universelle Prävention im Bereich FASD beinhaltet auch die Aufklärung und Schulung von ÄrztInnen, PsychologInnen, PädagogInnen und anderen Fachkräften. Eine Umfrage unter 428 Professionellen in Deutschland hatte zwar ergeben, dass über 95 % das Risiko der intrauterinen Alkoholexposition für die kindliche Entwicklung kannten, die Prävalenz mütterlichen Alkoholkonsums und der FASD jedoch deutlich unterschätzten. Auch im Bereich der Diagnostik und des Langzeitverlaufs von PatientInnen mit FASD zeigte sich in dieser Umfrage ein signifikanter Mangel an für die Versorgung der PatientInnen relevantem Wissen [24].

### Selektive Prävention

Die selektive Präventionsstrategie spricht explizit Frauen während der Schwangerschaft an, insbesondere Frauen mit riskanten Konsummustern oder Alkoholabhängigkeit *vor *der aktuellen bzw. in einer vorausgegangenen Schwangerschaft oder Frauen mit einem mit FASD geborenen Kind.

Die frühestmögliche Identifizierung von riskantem Alkoholkonsum vor und von jeglichem Alkoholkonsum in einer Schwangerschaft ist die wesentliche Voraussetzung für die Anwendung selektiver Präventionsinterventionen [16]. Als Messinstrument wird von der Deutschen Hauptstelle für Suchtanfragen die Verwendung des T‑ACE-Fragebogens (T = „tolerance“: Toleranzentwicklung, A = „felt annoyed“: anderen fiel der Alkoholkonsum als störend auf, C = „cut down“: eigener Versuch, Alkoholkonsum zu reduzieren, E = „eye opener“: morgendlicher Alkoholkonsum) empfohlen. Dabei ist zu berücksichtigen, dass dieser zwar aktuellen oder früheren riskanten Alkoholkonsum erhebt, die äußerst relevante Frage nach der Punktnüchternheit jedoch fehlt [25].

Wichtige Institutionen für die Implementierung selektiver (und universeller) Präventionsinterventionen sind die gynäkologischen Praxen sowie das eher pädagogisch ausgerichtete Setting der Schwangerschaftsberatungsstellen. Gynäkologische Praxen bieten das Potenzial, sowohl junge Mädchen frühzeitig als auch schwangere Frauen durch routinemäßige Ansprache und Information zu den Gefahren von Substanzkonsum in der Schwangerschaft zu sensibilisieren. Seit dem Jahr 2011 sollen ÄrztInnen Frauen im Rahmen der Schwangerschaftsvorsorgeuntersuchungen bei der Beratung über Genussmittel ausdrücklich auf Alkohol, Tabak und andere Drogen hinweisen und dies im Mutterpass vermerken. Im Praxisalltag scheint diese Beratung jedoch größtenteils nur sehr flüchtig durchgeführt zu werden – eine ausführliche Aufklärung findet laut vielen Frauen nicht statt. Bei einigen GynäkologInnen scheint weiterhin die (meist unbegründete) Angst zu bestehen, dass Fragen zum Konsumverhalten zum Vertrauensbruch und daraus resultierend zum Arztwechsel der schwangeren Frauen führen.

Für Frauen, die bereits ein Kind mit FASD geboren haben, spielen die betreuenden Kinder- und JugendärztInnen eine große Rolle: einerseits für die Früherkennung der FASD beim geborenen Kind und andererseits für die Unterstützung der Mutter hinsichtlich Alkoholreduktion oder -abstinenz in Folgeschwangerschaften.

### Indizierte Prävention

Die indizierte Präventionsstrategie richtet sich an die Hochrisikogruppe von Frauen mit fortbestehendem Alkoholkonsum nach Bekanntwerden der Schwangerschaft [25]. Fachkräfte müssen als Voraussetzung zur Umsetzung dieser Präventionsmaßnahme schwangere Frauen mit problematischem Alkoholkonsumverhalten erkennen (z. B. mittels medizinischer Anamnese oder des oben erwähnten T‑ACE-Fragebogens), dieses ansprechen und indizierte Interventionen anbieten oder extern vorhandene Hilfestellungen aufzeigen. Laut O’Connor et al. tragen Kurzinterventionen zu einer Reduktion des Alkoholkonsums in der Schwangerschaft bei [26]. Als zusätzliche Maßnahmen können intensive, kontinuierliche ambulante oder stationäre Suchttherapien notwendig sein [25]. Bei weiterhin bestehendem riskanten Alkoholkonsum in der Schwangerschaft empfiehlt die S3-Leitlinie „Screening, Diagnose und Behandlung alkoholbezogener Störungen“ das Angebot von Kurzinterventionen, psychotherapeutischen Interventionen und/oder psychosozialer Therapie [27]. Ein aktives, initiatives Zugehen der behandelnden Fachkräfte auf die schwangere Frau sowie eine wertschätzende, positive Beratungsbeziehung ohne Schuldzuweisung und Stigmatisierung mit dem Ziel der Alkoholabstinenz haben sich als sinnvolle Grundsätze erwiesen [25].

In Deutschland führten beispielsweise Schwangerschaftsberatungsstellen in Kooperation mit der Suchthilfe 2012 bis 2014 die Interventionsstudie „Kölner Interventionsmodell zur Prävention des Alkohol- und/oder Tabakkonsums in Schwangerschaft und Stillzeit“ durch. Das Ziel des Projektes waren die Erprobung und Entwicklung eines Stepped-Care-Modells. Das Interventionsmodell umfasste die Komponenten Screening zu Alkohol- und/oder Tabakkonsum, motivierende Kurzinterventionen und für ehemals oder aktuell Suchtmittel konsumierende Mütter von Kindern die Erziehungskompetenzförderung „Mehr MUT!“. In der 16-monatigen Untersuchungsphase nahmen insgesamt 1858 Frauen an der Intervention teil. Die Beratungszeit betrug im Durchschnitt 66 min, wobei das Gespräch zu Alkohol und Tabak durchschnittlich knapp 11 min in Anspruch nahm. Aus Sicht der SchwangerschaftsberaterInnen reagierten 82,6 % der teilnehmenden Frauen positiv und 2,6 % ablehnend auf das Screening zum Alkohol- und/oder Tabakkonsum. Auf motivierende Kurzinterventionen reagierten 70,0 % der Frauen positiv, 8,4 % zeigten eine ablehnende Reaktion [28].

Auch bei der indizierten Prävention sind GynäkologInnen und Hebammen/Entbindungspfleger wichtige Mitspieler für die Identifikation alkoholkonsumierender Schwangerer und für das Angebot zu Unterstützungsmaßnahmen bezüglich Abstinenz oder Alkoholreduktion.

Ergänzend können internetbasierte Präventionsangebote ortsunabhängige Alternativen und einen niedrigschwelligen Zugang bieten. Laut einer repräsentativen Studie des Digitalverbandes Bitkom verwendeten im Jahr 2019 bereits 65 % der befragten Smartphonenutzenden (Personen ab 16 Jahren) Gesundheits-Apps [29]. Auch schwangere Frauen informieren sich zunehmend über das Internet, häufig zu Beginn der Schwangerschaft [30, 31].

Die vom BMG geförderte IRIS-Plattform („individualisierte, risikoadaptierte internetbasierte Intervention zur Verringerung des Alkohol- und Tabakkonsums bei Schwangeren“) bietet Onlineberatung sowie interaktive Übungen im Internet zu den Themen Alkohol und Tabak in der Schwangerschaft. Teilnehmende sollen dadurch von der Information bis zum Konsumstopp begleitet werden. Die Module wurden basierend auf etablierten therapeutischen Programmen aufgebaut. In einem Zeitraum von 20 Wochen registrierten sich 25 Frauen im Tabak-, 4 im Alkohol- und eine im kombinierten Programm (Alkohol und Tabak). 3 Monate nach Programmbeendigung berichteten 18,5 % der Frauen im Tabakprogramm sowie 3 Frauen im Alkoholprogramm in der Selbstauskunft über eine Abstinenz. Aussagen zur subjektiven Zufriedenheit konnten lediglich von 6 Probandinnen erhoben werden, wobei die Möglichkeit des E‑Coachings mit einem Mittelwert von 1,9 positiv bewertet wurde (Noten von 1 = sehr gut bis 6 = ungenügend; [32]).

### Primär‑, Sekundär- und Tertiärprävention bei FASD

Die Einteilung in primäre, sekundäre und tertiäre Prävention erfolgt nach dem klassischen Präventionsmodell nach Caplan [33].

Die Primärprävention umfasst Maßnahmen zur Verhinderung des Auftretens einer Erkrankung. In Bezug auf FASD sind dies dementsprechend Motivationsmaßnahmen zum Verzicht auf Alkohol in der Schwangerschaft (wie teilweise bereits unter „universelle Prävention“ beschrieben).

Die Sekundärprävention soll dazu beitragen, Krankheiten frühzeitig zu erkennen und zu therapieren. Die Basis für eine frühzeitige, korrekte und in Deutschland einheitliche Diagnosestellung von FASD ist durch die Entwicklung und Implementierung der S3-Leitlinie „Fetale Alkoholspektrumstörungen – FASD – Diagnostik“ [4] geschaffen worden. Darauf aufbauend sollten, ebenfalls frühzeitig, adäquate Therapie- und Unterstützungsmaßnahmen für das Kind mit FASD und dessen Familie initiiert werden, die individuell je nach neuropsychologischem Profil geplant und im Entwicklungsverlauf adaptiert werden müssen. Als besonders relevant erwies sich außerdem die Einbeziehung von Elterntrainings und die konstante langfristige Betreuung und Unterstützung der Familien [34].

Unter Tertiärprävention versteht man unterstützende Maßnahmen, welche mögliche Folgeschäden bzw. Sekundärerkrankungen oder die Chronifizierung einer Erkrankung vermeiden oder zumindest vermindern sollen. Im Jugend- und Erwachsenenalter können Sekundärerkrankungen wie eigenes Suchtverhalten und andere psychiatrische Störungen durch protektive Faktoren, wie ein stabiles Umfeld im Kindesalter, eine frühe Diagnose und eine gewaltfreie Erziehung und Umwelt, vermieden oder zumindest deren Auftretenswahrscheinlichkeit reduziert werden [7, 11]. In einer US-amerikanischen Studie wurden Eltern von Kindern mit FASD und Fachkräfte, die Kinder mit FASD versorgen, befragt [35]. Dabei wurden folgende Barrieren auf systemischer Ebene identifiziert, die zum Auftreten von Sekundärerkrankungen bei FASD beitragen: eine späte Diagnosestellung, das Fehlen von geeigneten Therapiemaßnahmen und fehlende Fortführung von effektiven Interventionen im Krankheitsverlauf. In einer weiteren qualitativen Datenerhebung konnten 5 für die Prävention von Sekundärerkrankungen von FASD wichtige Eigenschaften für Interventionen festgelegt werden. Die Interventionsprogramme sollten:

1. lebenslang zugänglich sein,
2. sich auf präventive Ansätze fokussieren,
3. individuell angepasst werden,
4. einen interdisziplinären, umfassenden therapeutischen Ansatz aufweisen und
5. dem Entwicklungsstand entsprechen [36].

Aus der klinischen Erfahrung kann sich eine kontinuierliche Anbindung an ein FASD-erfahrenes Zentrum, welches die bedarfsorientierten Förder- und Therapiemaßnahmen von Kindern und Jugendlichen mit FASD koordiniert und ggf. anpasst, positiv auf deren Entwicklungsverlauf und ihre Lebensqualität innerhalb des Familienverbundes auswirken (z. B. das Projekt Deutsches FASD KOMPETENZZENTRUM Bayern, www.deutsches-fasd-kompetenzzentrum-bayern.de).

## Fazit

Die Fetale Alkoholspektrumstörung (FASD) ist eine sehr häufige und dabei leider sehr häufig übersehene, bei Geburt bestehende, chronische Erkrankung. Sie führt zu deutlichen Beeinträchtigungen der betroffenen Kinder, Jugendlichen und Erwachsenen in der Entwicklung, Kognition und selbstständigen Lebensführung. Aktuell wird davon ausgegangen, dass durch Alkoholverzicht in der Schwangerschaft die FASD vermieden werden kann. Daher kommt, neben der Sekundärprävention in Form von frühzeitiger Diagnose und Tertiärprävention durch adäquate, kontinuierliche Versorgung der bereits erkrankten Kinder, der Primärprävention eine sehr große Bedeutung zu. Alle Präventionsmaßnahmen sollten strukturiert und flächendeckend, basierend auf der Kooperation von Medizin und Politik, geplant und wissenschaftlich begleitet durchgeführt werden. Unser Ansatzpunkt in der (Neuro‑)Pädiatrie ist dabei die Aufklärung von ÄrztInnen und anderen medizinischen Fachkräften.
